# Safety and Effectiveness of Copaiba Oleoresin (*C. reticulata* Ducke) on Inflammation and Tissue Repair of Oral Wounds in Rats

**DOI:** 10.3390/ijms21103568

**Published:** 2020-05-18

**Authors:** María Olimpia Paz Alvarenga, Leonardo Oliveira Bittencourt, Paulo Fernando Santos Mendes, Julia Turra Ribeiro, Osmar Alves Lameira, Marta Chagas Monteiro, Carlos Augusto Galvão Barboza, Manoela Domingues Martins, Rafael Rodrigues Lima

**Affiliations:** 1 Laboratory of Functional and Structural Biology, Biological Sciences Institute, Federal University of Pará, Belém 66075-110, Brazil; mop.alvarenga@gmail.com (M.O.P.A.); leo.bittencourt25@gmail.com (L.O.B.); paulofsmendes@gmail.com (P.F.S.M.); 2 Faculty of Department of Oral Pathology, School of Dentistry, Federal University of Rio Grande do Sul, Porto Alegre 90040-060, Brazil; juliaturraribeiro@gmail.com (J.T.R.); manomartins@gmail.com (M.D.M.); 3 Laboratory of Biotechnology, Embrapa Amazônia Oriental, Belém 66075-110, Brazil; osmar.lameira@embrapa.br; 4 Laboratory of In Vitro Tests, Immunology and Microbiology, Health Science Institute, Federal University of Pará, Belém 66075-110, Brazil; martachagas2@yahoo.com.br; 5 Department of Morphology, Federal University of Rio Grande do Norte, Natal 59078-900, Brazil; carlosaugusto2000@hotmail.com

**Keywords:** copaiba oleoresin, wound healing, alternative therapy, natural anti-inflammatory, *C. reticulata* Ducke

## Abstract

In traditional communities of the Brazilian Amazon, the copaiba oleoresin (*C. reticulata* Ducke) is widely known for its therapeutic activity, especially its wound healing and anti-inflammatory actions. Our study aimed to evaluate these effects in oral lesions and the safety of the dosage proposed. A punch biopsy wound was induced on the ventral surface of the tongue of forty-five male *Wistar* rats under anesthesia. Animals were randomly allocated to one of three groups based on the treatment: control, corticoid and copaiba. A daily dose of each treatment and vehicle was administrated by oral gavage for three consecutive days. Sample collections took place on the third, seventh and 15th days post-wounding for clinical and histopathological analyses. Blood was collected on the third and seventh days for kidneys and liver function tests. Semi-quantitative analyses were performed based on scores of inflammation and reepithelization. Tissue collagen deposition was detected by PicroSirius red staining. Copaiba-treated wounds revealed a smaller wound area, decreased of acute inflammatory reaction and enhanced reepithelization. The levels of kidney and liver function tests did not reveal presence of damage post-treatments. Our findings suggest that copaiba oleoresin is a safe and effective alternative therapy for inflammation and tissue repair of oral wounds in this animal model.

## 1. Introduction

Aphthae and traumatic ulcers are common inflammatory conditions in the oral mucosa. These oral lesions are painful and produce discomfort during food intake or even speaking, interfering with the patient’s quality of life [1,2]. Although there is no a standard treatment, management is based on widely used anti-inflammatory therapy—such as corticoids—generally prescribed by dental consultant [3,4]. However, corticoids therapy has been associated with a delay in the reepithelization of open wounds and high doses and long-term therapy with renal and liver damages [5,6]. Therefore, it is important to introduce alternative therapies in oral lesions. 

The use of medicinal plants for healing is the oldest form of medicinal practice of mankind [7]. Contemporary science has shown that some natural compounds have high anti-inflammatory, antioxidant and regenerative effects [8]. Brazil has great biodiversity (15–20% of the world’s biodiversity), with about 60,000 varieties of plants, including some known for traditional use and others still not explored [9]. Although the main advantages of using remedies of plant origin are the low cost and usually smaller number of side effects [10], they are composed of several substances in addition to the active compound which can have synergistic or antagonistic action [11]. Thus, it is important to study its effectiveness as well as its safety in medical applications.

Traditional communities in the Brazilian Amazon have reported the anti-inflammatory, analgesic, and wound healing effects of copaiba oleoresin [12]. Copaiba oleoresin is extracted from the trunk of the genus copaifera tree, which is the most commonly used *C. reticulata* Ducke species. This oleoresin has resinous acids and volatile compounds in its chemical composition, such as diterpenes and sesquiterpenes. The predominant forms are β-Caryophyllene (bactericidal and anti-inflammatory), β-Bisabolene (anti-inflammatory) and α-Humulene (anti-inflammatory)—present in several essential oils [13,14]. β-Caryophyllene is a ligand of the cannabinoid receptor 2 (CB_2_); its activation has been associated with decreasing pain, a major signal of inflammatory response, and enhancing reepithelization [15,16]. The α-Humulene has been related with the promotion of angiogenesis, helpful in wound healing [17]. 

Previous studies revealed the effectiveness of copaiba oleoresin in modulating inflammatory infiltrate in oral lesions compared to corticoid-treated [14,18]. However, the effect on reepithelization and the safety of the dosage administrated concerning renal and liver functions, have not been assessed. Thus, this study aimed to investigate the therapeutic effects of copaiba oleoresin (*C. reticulata* Ducke) on reepithelization by decreasing inflammatory response in an animal model of traumatic ulcer induced in the tongue of rats. In addition, we sought to analyze the safety of the dosage used in this experiment through analyses of biochemical parameters of liver and kidneys functions in order to introduce the oleoresin as an alternative therapy in oral lesions.

## 2. Results

### 2.1. Copaiba Oleoresin Accelerates the Wound Contraction

**Figure 1:**
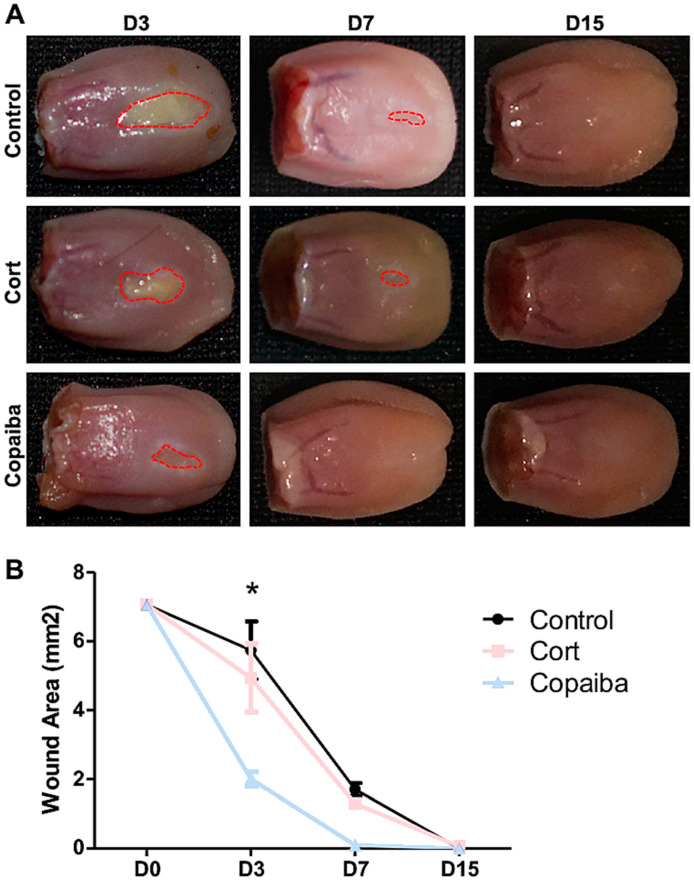
Clinical analysis of the wound area post-wounding. (**A**) Wounds post-treatment with copaiba oleoresin (200 mg/kg/day) and corticoid–dexamethasone (0.5 mg/kg/day), and untreated wounds. Copaiba-treated ulcers presented smaller wound area on D3 and completely disappeared in D7. Corticoid treatment promoted a reduction of the wound area on D3 compared to controls, but less effective than the copaiba-treated group. (**B**) Graph comparing the contraction of the wounds throughout the time at D3 (*n* = 15), D7 (*n* = 15) and D15 (*n* = 15) in untreated and treated groups. Two-way analysis of variance (ANOVA) followed by Tukey’s multiple comparison tests (* *p* < 0.05).

The clinical analysis demonstrated that copaiba oleoresin promoted an early reduction of the wound area when compared to corticoid and control (Figure 1). The wound area on day 3 (D3) post-wounding showed significant difference between groups, in which the copaiba-treated wounds presented a significantly smaller area compared to the corticoid-treated group (*p* < 0.05) and controls (*p* < 0.01) (Figure 1A). On day 7 (D7), wounds had completely disappeared in the copaiba-treated group. Animals from all groups presented complete wound closure (area = 0 mm^2^) on day 15 (D15). 

### 2.2. Copaiba Oleoresin Modulates Inflammatory Process and Accelerates Reepithelization 

**Figure 2:**
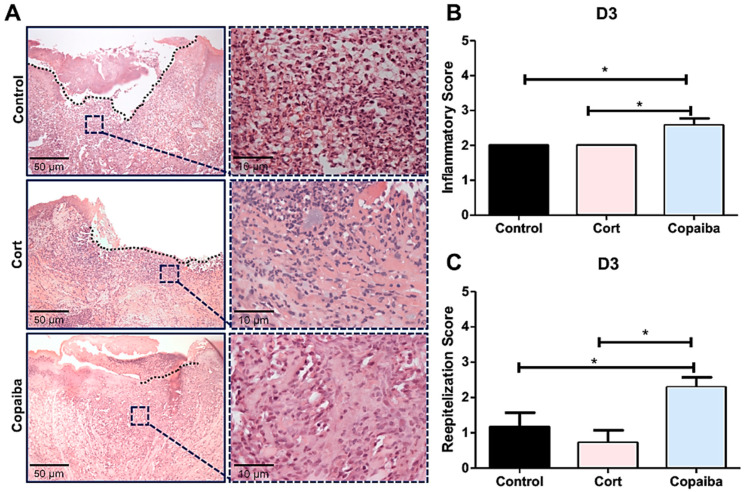
Histopathological analysis of treated and untreated wounds at D3. (**A**) Five-μm sections stained by hematoxylin and eosin (HE) showing copaiba-treated wounds with predominance of granulation tissue, whereas acute inflammatory cells (neutrophils) predominated in most wounds from the corticoid and control groups. (**B**) Copaiba-treated wounds presented a significantly higher score of the inflammatory stage, which indicates a decreased acute inflammatory reaction and (**C**) a more advanced stage of reepithelization. The corticoid-treated wounds showed lower scores of reepithelization at D3, demonstrating poor tissue repair (original magnification ×200 and ×1000). One-way ANOVA and Tukey’s post hoc tests (*n* = 15) (* *p* < 0.05).

**Figure 3:**
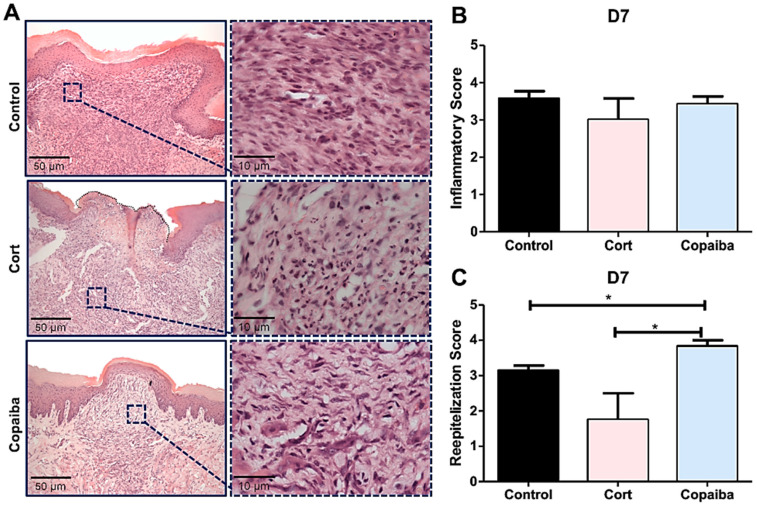
Histopathological analysis of treated and untreated wounds at D7. (**A**) Five-μm sections stained by HE, showing complete closure of the wound with a uniform thickness in most of the copaiba-treated samples analyzed compared to the corticoid-treated and control samples. (**B**) Inflammatory scores presented no significant differences between groups on this day. (**C**) Copaiba-treated wounds presented an advanced reepithelization stage. The corticoid-treated group still revealed a poor reepithelization performance on this day (original magnification ×200 and ×1000). One-way ANOVA and Tukey’s post hoc tests (*n* = 15) (* *p* < 0.05).

The semi-quantitative analysis revealed significant differences in inflammatory and reepithelization scores between groups on D3 (* *p* < 0.05) (Figure 2B) and no significant differences on D7 (Figure 3B). Copaiba-treated wounds presented significant higher scores (more advanced inflammatory stage which indicates decreased acute inflammatory reaction) on D3 (Figure 2B) and higher reepithelization scores (more advanced stage of tissue repair) on D3 and D7 compared to other groups (* *p* < 0.05) (Figure 2C and Figure 3C).

Although all the groups presented open wounds on D3, differences in connective tissue were observed among the groups. Copaiba-treated wounds presented recruitment of inflammatory cells such as predominance of granulation tissue (lymphocytes, macrophages, collagen and new vessels) in most of the samples analyzed (Figure 2A,B). Therefore, higher reepithelization scores were observed at an early time point when compared to corticoid-treated and control groups (Figure 2C). Acute inflammatory cells (neutrophils) predominated in most wounds from the corticoid and control group on this day (Figure 2A).

The resolution of the inflammatory stage in the connective tissue characterized by the disappearance of chronic inflammation was observed in all the groups on D7 (Figure 3A), though with some differences in collagen deposition.

### 2.3. Copaiba Oleoresin Promoted Collagen Formation

**Figure 4:**
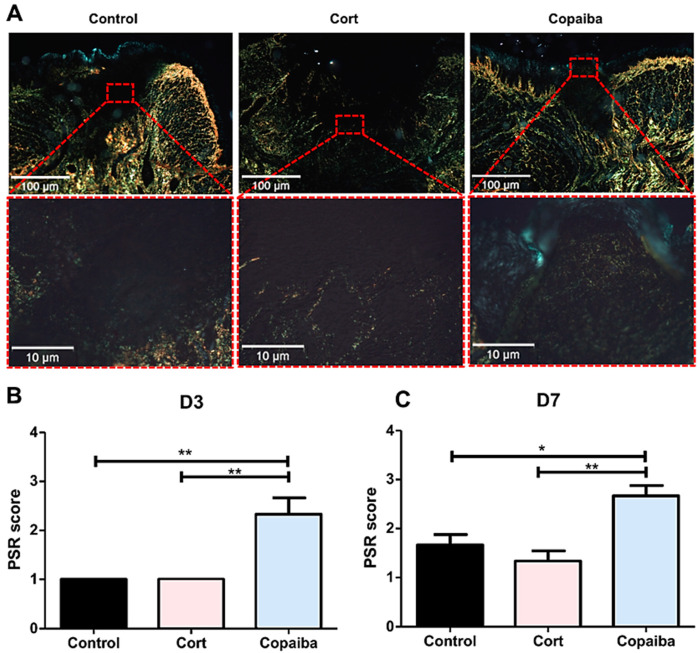
Photomicrographs of PicroSirius red staining. (**A**) Corticoid-treated and controls’ wound area showed a black area (especially on the surface), indicating the absence or a small number of collagen fibers. On the side and bottom of the wound, poorly organized thin collagen fibers were observed. Copaiba-treated wounds were filled with more organized collagen fibers. (PSR, original magnification, ×100 and ×400). (**B**) Graphs of collagen content analysis on D3 (*n* = 15) and (**C**) D7 (*n* = 15). Note that on D3 and D7, corticoid-treated and control groups showed less organized fibers than the copaiba-treated group (original magnification ×100 and ×400). One-way ANOVA followed by Tukey’s multiple comparison tests (* *p* < 0.05, ** *p <* 0.01).

In the PicroSirius red staining on D3, the control and corticoid-treated groups revealed areas with no red or green birefringence, indicating the absence of collagen fibers and part of the wound with thin, delicate, loosely arranged collagen fibers (+1 score). Copaiba-treated wounds revealed thin, delicate, loosely arranged collagen fibers in some areas and thicker and gross fibers in other areas of the wound in most samples analyzed (+2 score) (Figure 4). The collagen was significantly better organized in the copaiba-treated than in corticoid and control groups (** *p* < 0.01) (Figure 4A). 

On D7, corticoid-treated and control wounds displayed thin collagen fibers, which were poorly organized. In the groups treated with copaiba, some denser collagen fibers with a thicker appearance were noted in the wound. These fibers were more parallel to the epithelium and demonstrated a more organized pattern in comparison to the control (* *p* < 0.05) and corticoid group (** *p* < 0.01). (Figure 4C)

### 2.4. The Dosage of Copaiba Administered Concluded to Be Safe for Liver and Kidneys

**Figure 5:**
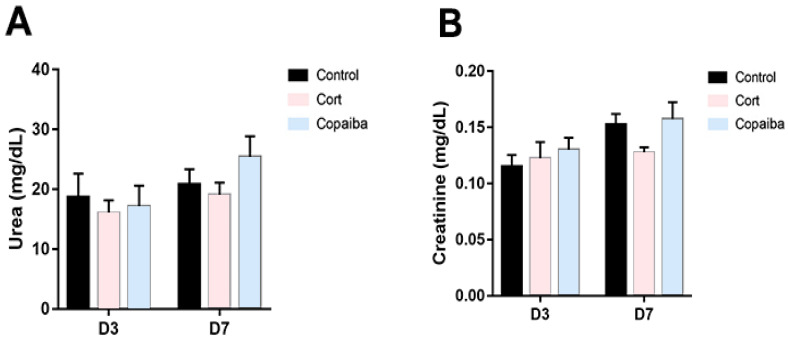
Levels of renal function parameters in different groups. (**A**) No significant differences between the groups were found in the levels of urea and (**B**) creatinine in plasma at D3 (*n* = 15) and D7 (*n* = 15), suggesting that the dosage of both copaiba and corticoid administered was harmless for the kidneys. The results are expressed as the mean ± standard error of the mean. One-way ANOVA and Tukey’s post hoc tests.

The levels of biochemical parameters of renal and liver functions are commonly increased in blood plasma due to the presence of different diseases or as a signal of damage to the liver or kidneys [5,19,20]. In this regard, the serum levels of urea (Figure 5A) and creatinine (Figure 5B) did not reveal significant differences between copaiba-treated animals and the other groups after the dosage administrated by oral gavage for three consecutive days.

**Figure 6:**
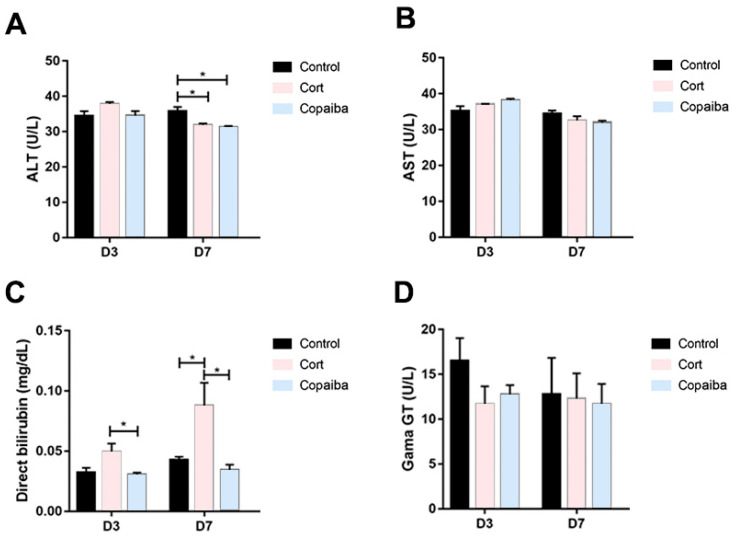
Comparative biochemical parameters levels of liver function in different groups: (**A**) ALT, (**B**) AST, (**C**) DB and (**D**) GGT (* *p* < 0.05). Although DB was elevated in the corticoid-treated group on D3 (*n* = 15) and D7 (*n* = 15) (Figure 6C), GGT levels presented normal levels in all the groups. The results are expressed as the mean ± standard error of the mean. One-way ANOVA and Tukey’s post hoc tests (* *p* < 0.05).

The serum levels of alanine aminotransferase (ALT) were significantly decreased in copaiba and corticoid-treated at the D7 and there were no differences between the groups at the D3 (Figure 6A). No significant differences were found in aspartate aminotransferase (AST) values between the groups (Figure 6B). The direct bilirubin (DB) values were significantly increased in the corticoid-treated compared to the copaiba-treated and control groups (Figure 6C). No significant differences were found in the serum levels of gamma-glutamyl transferase (GGT) between the groups (Figure 6D). 

## 3. Discussion

Despite oral mucosa wounds being able to heal more rapidly than skin wounds [21], some oral lesions may produce discomfort during food intake or even speaking, interfering with the patient’s quality of life, making effective wound healing therapy necessary [1,2]. The results of this animal model study demonstrate the safety and effectiveness of copaiba oleoresin (*C. reticulata* Ducke) on the wound-healing process in oral mucosa compared to corticoid therapy—often prescribed by dental professionals for oral lesions [3,4]. The systemic administration of the oleoresin promoted an anti-inflammatory effect at an early time point, accelerating the wound resolution when compared to untreated and corticoid-treated wounds, in which a poor tissue repair was observed. Moreover, the biochemical analyses revealed that the dosage of Copaiba oleoresin administered in our experiment was harmless to the kidneys and liver of *Wistar* rats.

Wound healing occurs in three overlapping phases: inflammatory, proliferative and remodeling [22], although it is more accelerated in oral mucosa wounds than cutaneous wounds [21]. In either case, the inflammatory reaction is the earliest event to take place through neutrophils and macrophages infiltrations and proliferation promotion [23]. Although our clinical analysis showed a reduction of wound area in corticoid-treated on the third day post-wounding (Figure 1A), the connective tissue still demonstrated the predominance of acute inflammatory cells (neutrophils) in most wounds from the corticoid-treated as well as untreated groups, whereas in the copaiba-treated group, an advance inflammatory stage with less neutrophils and the predominance of granulation tissue such as macrophages, lymphocytes, collagen and new vessels were observed, suggesting an accelerated tissue repair (Figure 2A). It has been determined that macrophages play an important role in the acceleration of tissue repair [23]. Apparently, the corticoid promoted an anti-inflammatory effect on the first days post-wounding; nonetheless, the poor wound healing performance observed from the third day onwards suggests an adverse effect delaying reepithelization (Figure 2C, Figure 3C and Figure 5). These results agree with previous studies in which it was reported that corticoid therapy might delay reepithelization by inhibiting the growth of new vessels (angiogenesis), although the mechanism through which this inhibition occurs has not been stablished [24].

The wound healing activity of the copaiba oleoresin can be mainly attributed to the anti-inflammatory activity of the β-Caryophyllene—since it is commonly the predominant compound found in oleoresins from the copaifera species [13] but also to the synergetic activity of the β-Caryophyllene with the α-Humulene [25]—a compound found in the *C. reticulata* Ducke [13]. β-Caryophyllene is a ligand of the cannabinoid receptor 2 (CB_2_) presented in various essential oils and its activation has been associated with decreasing pain, a major signal for inflammatory response, and enhancing reepithelization through multiple routes [16]. The CB_2_ receptors are involved in stimulating the secretion of pro-inflammatory cytokines and increasing T and B lymphocyte response. Cells treated in vitro with β-Caryophyllene have shown a reduction in the inflammatory phase and an early activation of the proliferative phase [13,14,15]. This proliferative phase consists of four fundamental tissue modifications: reepithelization, formation of new vessels, granulation tissue formation and collagen deposition [16]. The α-Humulene has been involved in the secretion of Interleukin 8 (IL-8), a chemokine produced by macrophages, which has various functions, including promoting the formation of new vessels [17]. In one study, the β-Caryophyllene was synergistic with α-Humulene, potentiating the secretion of IL-8 [25], being helpful in the proliferative phase. At the beginning of this phase, the collagen fibers present a disorganized pattern; subsequently, new fibers are produced and deposited in a more organized way following the adjacent connective tissue, initiating the remodeling phase [23,26]. Concerning this, our results revealed that oleoresin might accelerate the remodeling phase since the collagen was significantly better organized in copaiba-treated groups than in the corticoid-treated and control groups, in which less organized fibers were observed (Figure 4). Furthermore, the histological analyses also show an advanced reepithelization in the copaiba-treated group with a complete closure of the wound with a uniform thickness in most samples analyzed on the seventh day. In contrast the corticoid-treated group presented an open wound with a new oral mucosa lining at the wound edges with an irregular thickness, even worse than the untreated, in which reepithelization covering the entire wound was observed, though with irregular thickness (Figure 3A).

In a previous study of our group [18], the anti-inflammatory effect of systemic administration of copaiba oleoresin (*C. reticulata* Ducke) was assessed in transfixing injuries of tongues in *Wistar* rats and also revealed a modulation of the inflammatory response, similarly to the present study. However, the enhancement of reepithelization by this modulation was not evaluated because the wounds crossed the muscular plane and the wound resolution could not be observed at the end of the experiment [18]. Another study showed that 200 and 400 mg/kg body weight of copaiba oleoresin reduced neutrophilic infiltration and colonic mucosal damage following acetic-acid-induced colitis in rats [27]. The same attenuation of neutrophil recruitment was found following acute damage to the central nerve system, in which the oleoresin acted as an anti-inflammatory and neuroprotective agent [28]. Nonetheless, when oleoresin effectiveness was evaluated by topical application in oral lesions, it failed to accelerate wound healing [14]. Therefore, it may be suggested that copaiba oleoresin is only effective by systemic administration in wound healing therapy, probably due to the better absorption of its volatile compounds.

A recent study demonstrated that the oral cavity itself is primed for wound repair. Priming allows to rapidly control and limit inflammatory responses, leading to a faster wound closure compared to cutaneous wound healing. The gene expression signature changes during oral mucosal were analyzed in that study to identify and explain the mechanisms that define accelerated oral wound healing and it presented evidence that transcriptional regulators that define oral keratinocytes, such as SOX2 (sex-determining region Y-box 2) and PITX1 (paired-like homeodomain 1), hold the key to the activation of the molecular events responsible for accelerated wound resolution in oral healing [21]. These findings open a gap for future studies to identify the specific pathways activated by the copaiba oleoresin and their potential to accelerates wound repair and tissue regeneration in oral mucosa and furthermore, to understand the mechanism through which corticoids delay reepithelization.

Concerning the dosage of copaiba oleoresin, it is possible to indicate a safety margin for its oral administration as a therapeutic agent, since the LD50 was estimated to be greater than 2000 mg/kg body weight and classified as category 5, according to the Globally Harmonized System for the Classification and Labeling of Chemicals (GHS) [29]. However, all substances can cause harm at a certain level and it has been reported that taking high doses of copaiba can cause symptoms of intolerance, nausea, vomiting, colic and diarrhea, and prolonged use may cause renal and liver damages [29]. On the other side, although low doses of corticoids are considered safe, high doses of corticoid therapy have been also associated with renal and liver damages [6,30]. Therefore, a standard dosage of dexamethasone considered safe for the liver and kidneys was administered in this experiment (Table 1) [5,30]. In this regard, the dosage of copaiba oleoresin and corticoid used in our study was concluded to be in safe for the kidneys, since our biochemical analyses show normal levels of creatinine and urea in the plasma of all animals (Figure 5A,B). When assessing liver damage, our results show that the levels of ALT in the plasma of both copaiba and corticoid-treated were normal on the third day and decreased on the seventh day (Figure 6A). However, the values of conjugated bilirubin were significantly increased in the corticoid-treated on the third and seventh days (Figure 6C). Despite the elevated values of DB in the corticoid-treated group, the levels of GGT in plasma show normal values in all the groups (Figure 6D). Therefore, the higher values of conjugated bilirubin in the blood of the corticoid-treated groups may indicate a temporary biliary obstruction but without a risk of permanent damage considering that a safe dosage of corticoid was used in this experiment. Moreover, the dosage of copaiba administered in this study showed no damage signals in liver nor kidneys in the biochemical assessment (Figure 5 and Figure 6). 

Taken together, the aforementioned findings demonstrate that the dosage of copaiba oleoresin (*C. reticulata* Ducke) administered by oral gavage in our study were harmless to the liver and renal functions, and is a more effective therapy for inflammation and tissue repair of oral wounds compared to corticoid therapy. Oleoresin modulated the inflammatory infiltrate and promoted a faster resolution and closure of the wound with higher levels of indexes of reepithelization and better organization of the collagen fibers’ deposition. Nonetheless, more studies are needed to identify the specific pathways activated by the copaiba oleoresin and their potential to accelerate wound repair and tissue regeneration in human oral mucosa in order to introduce it as an alternative therapy.

## 4. Materials and Methods 

### 4.1. Study Design

This study was a prospective, randomized, controlled and blinded animal model study based on the Guide for the Care and Use of Laboratory Animals and the Animal Research: Reporting of In Vivo Experiments (ARRIVE) guidelines [31].

### 4.2. Plant Material, Characterization and Acute Oral Toxicity Test

The oleoresin was the same sample as that used in previous studies [18,28], collected and characterized by researchers from the Brazilian Agricultural Research Corporation (EMPRAPA). The extraction of the oleoresin was made by artificial exudation from the trunk of a native tree *C. reticulata* Ducke located in the municipality of Belterra, Pará, Brazil, approximately 30 years old. After collection, the oleoresin was stored in the absence of light, oxygen and heat in order to keep its volatile compounds stabilized. A sample of the plant was placed at the IAN EMBRAPA Herbarium (Exsiccate: 183,939). The characterization by gas chromatography–mass spectrometry (GC–MS) of the sample used was described by Santos-Guimarães et al., 2012 (Table A1).

The dose of the oil administered was based on the acute oral toxicity test that was carried out in the previous study according to Guide 425/2008 of the Organization for Economic Cooperation and Development (OECD) [18]. The lethal dose that kills 50% of test animal population (LD50) was estimated using the limit dose test (2000 mg/kg body weight). An amount of 10% of the lowest dose that kills at least three animals was selected, i.e., 200 mg/kg body weight [18,29].

### 4.3. Animals and Experimental Protocol

#### 4.3.1. Animals

Forty-five male *Wistar* rats (*Rattus novegicus*)—sample size based on test power from previous study [18]—with body weights of approximately 200–250 g and 60 days old, were cared under aseptic conditions and housed individually in polypropylene cages in a climate-controlled room (12 h light/dark cycle and 25 ± 1 °C) with food and water ad libitum.

The experimental protocol was carried out with the rules issued by the National Council for Animal Control and Experimentation (CONCEA) and approved by the Ethics Committee on the Use of Animals (CEUA) of the Federal University of Pará (UFPA) under the license number 6534071217, on 01/18/2018.

#### 4.3.2. Experimental Groups and Wounding Method 

On the procedure day, animals were anesthetized by intraperitoneal injection of ketamine hydrochloride (90 mg/kg) and xylazine hydrochloride (10 mg/kg). After checking for loss of reflexes, animals were placed on the operating table in the supine position and with the oral cavity open. The tongue was pulled out with a clinical smooth press tack tweezer, exposing the ventral surface and was cleaned with 2% chlorhexidine before procedure.

A traumatic ulcer was induced in the ventral surface 5 mm from the apex and at the midline with a standard technique using a disposable biopsy punch of circular format with a diameter of 3 mm. The punch was pressed into the tissue to penetrate to 2-mm deep without crossing the muscle plane. All wounds were induced by a single operator trained in a pilot study.

A random allocation into three groups was performed based on body weight and the type of treatment to be received was identified as one of the following: control, corticoid and copaiba (Table 1).

**Figure 7:**
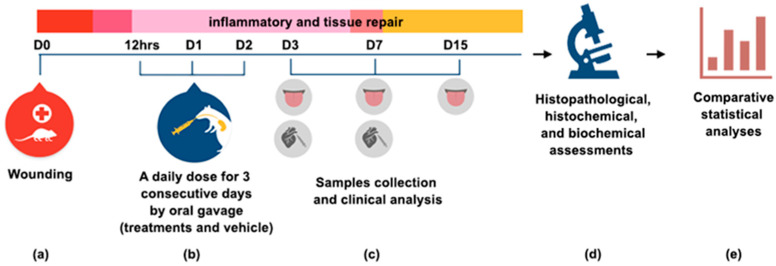
Methodological summary of the experiment (**a**) On day zero (D0), a traumatic ulcer in the ventral surface of tongue was induced with a standard punch biopsy technique (diameter 3 mm). (**b**) Treatments were administrated 12 h post wounding, and the next two days by oral gavage. (**c**) On D3, D7 and D15, samples were collected for clinical, histopathological and histochemical analysis. Blood was collected by cardiac puncture for a biochemical assessment of liver and kidneys functions at D3 and D7. (**d**) The different analyses were performed and (**e**) the data were tabulated for a comparative statistical analysis. The results are expressed as the mean ± standard error of the mean.

The copaiba oleoresin was mixed with saline, as an emulsion dilution, and Tween 20 at 5% to enable the solubilizing of the oil in saline. A daily dose of the treatments and vehicle was administered by oral gavage 12 h after induction of the traumatic ulcer and the next two days (D1 and D2). A methodological summary of the experiment is shown in Figure 7.

In euthanasia days, five animals per group were anesthetized for sample collection. The blood was collected by cardiac puncture and transferred to heparinized tubes. Then, animals were euthanized by cervical dislocation for the dissection of the tongue.

### 4.4. Clinical Analysis

The animals were weighed on the surgery day (day 0) and euthanasia days (day N). Wounds were photographed next to a millimeter ruler. Wound area percentage was calculated by the following formula:

Wound area (%) = [Area (day N)/Area (day 0)] × 100(1)

The wound area was calculated in pixels using ImageJ 1.48v software (National Institutes of Health, USA). Then, the values were converted to mm^2^ using the ruler as a scale reference. The clinical analysis was blinded.

### 4.5. Histopathological ANALYSES

After photographs were taken, the tongues were cut at their ends—to facilitate the penetration of the fixative—and immediately immersed in a 10% buffered formalin solution (10 mM phosphate buffer, pH 7.4) for 72 h. The samples were dehydrated in an alcoholic battery (70–100%), immersed in xylol solution for clarification and subsequently, were embedded in paraffin wax, cut into 5-μm sections and then stained with HE. Two experienced pathologists performed a blind semi-quantitative evaluation based on scores. 

The inflammatory process was analyzed based on scores in which: Grade 1—acute inflammation (pyogenic membrane); Grade 2—predominance of diffuse acute inflammation; Grade 3—predominance of chronic inflammatory process; Grade 4—resolution and healing (reduction or disappearance of chronic inflammation) [32]. Reepithelization was also evaluated based on scores in which: Grade 0—reepithelization at the end of the wound; Grade 1—reepithelization covering less than half the wound; Grade 2—reepithelization covering more than half of the wound; Grade 3—reepithelization covering the entire wound with irregular thickness; Grade 4—reepithelization covering the entire wound and of normal thickness [33].

### 4.6. PicroSirius Red Staining

Tissue sections (5 µm) were stained with PicroSirius red staining and the analyzes were performed according to the intensity and pattern of the collagenization and the disposition of collagen fibers deposited at the wound site. The collagen fibers were classified into +1 (thin, delicate, loosely arranged collagen fibers seen throughout the wound area), +2 (thin, delicate, loosely arranged collagen fibers in some areas and thicker and gross fibers in other areas of the wound) and +3 (thick, gross, densely arranged collagen fibers seen throughout the wound area) [34]. The pattern of collagen arrangement of normal mucosa was used to define the score +3.

The scores of the samples were defined in the polarized images by one pathologist and a descriptive analysis of each group was performed. A polarized microscope (Olympus BX51, Tokyo, Japan) coupled with a camera device (Olympus Q-color 5 RTV, Tokyo, Japan) and capture software (Q-capture, version 2.0.11) were used at ×100 and ×400 magnification to acquire the images.

### 4.7. Analysis of Selected Biochemical Parameters of Liver and Kidney Functions

Blood samples were centrifuged at 3000 rpm for 10 min and the plasma was collected and distributed in microtubules, and then stored in an ultrafreezer (−80 °C). The following plasma levels of liver function were assessed: ALT, AST, DB and GGT. The plasma levels of creatinine and urea were assessed to analyze renal function. All the tests were performed according to standard procedures facilitated by the manufacturer (Bioclin, Brazil).

### 4.8. Statistical Analysis 

Statistical analyses were carried out using GraphPad Prism 6 (GraphPad Software, San Diego, CA, USA). Clinical analyses were performed by a two-way analysis of variance (ANOVA). Histopathological and PSR analyses were performed by a one-way analysis of variance (ANOVA). All analyses were followed by Tukey’s multiple comparison test. The asterisks denote statistical significance (* *p* < 0.05; ** *p* ≤ 0.01; *** *p* ≤ 0.001; and not significant (NS) *p* ≥ 0.05).

## Figures and Tables

**Table 1:** Experimental groups according to the doses of treatment or vehicle received.

Treatment Group (Sample Size)	Doses Administered by Oral Gavage
Control (*n* = 15)	200 mg/kg/day of saline solution and Tween 20 at 5%
Corticoid (*n* = 15)	0.5 mg/kg/day of Dexamethasone
Copaiba (*n* = 15)	200 mg/kg/day of Copaiba oleoresin

## Abbreviations

MED	Minimum Effective Dose
CTG	Corticoid Group
COG	Copaiba Group
CG	Control Group
D3	Day 3
D7	Day 7
D15	Day 15
HE	Hematoxylin and Eosin
DL50	Median Lethal Dose
GC-MS	Gas Chromatography - Mass Spectrometry
OECD	Organization for Economic Cooperation and Develpment
UFPA	Federal University of Pará
CEUA	Ethics Committee on the Use of Animals
ARRIVE	Animal Research: Reporting of In Vivo Experiments
CONCEA	National Council for Animal Control and Experimentation
ALT	Alanine Aminotransferase
AST	Aspartate Aminotransferase
CAPES	Coordination for Improvement of Higher Education Personnel
CNPq	Brazilian National Council for Scientific and Technological Development
EMBRAPA	Brazilian Agricultural Research Corporation
PROCAD	National Academic Cooperation Program

## Appendix A

**Table A1:** Chemical composition of the oleoresin volatiles of tree *C. reticulata* Ducke by GC-MS described by Santos-Guimarães et al. [28].

Constituents	%
δ-Elemene	0.2
Cyclosativene	0.9
α-Copaene	0.5
β-Elemene	3.3
β-Caryophyllene	37.6
trans-α-Bergamotene	9.3
Aromadendrene	0.9
epi-β-Santalene	0.1
α-Humulene + (E)-β-farnesene	5.3
β-Chamigrene	0.9
γ-Gurjunene	0.6
γ-Curcumene	0.6
β-Selinene	4.9
α-Selinene	3.1
(Z)-α-Bisabolene	1.8
α-Bulnesene	2.1
β-Bisabolene	13.9
β-Curcumene	0.4
β-Sesquiphellandrene	1.1
(E)-γ-Bisabolene	1.3
Caryophyllene oxide	0.2
epi-β-Bisabolol	0.1
β-Bisabolol	0.2
